# Impact of Reconstruction Route on Postoperative Morbidity After Esophagectomy: Analysis of Esophagectomies in the Japanese National Clinical Database

**DOI:** 10.1002/ags3.12501

**Published:** 2021-09-06

**Authors:** Hirotoshi Kikuchi, Hideki Endo, Hiroyuki Yamamoto, Soji Ozawa, Hiroaki Miyata, Yoshihiro Kakeji, Hisahiro Matsubara, Yuichiro Doki, Yuko Kitagawa, Hiroya Takeuchi

**Affiliations:** ^1^ Department of Surgery Hamamatsu University School of Medicine Hamamatsu Japan; ^2^ Department of Healthcare Quality Assessment The University of Tokyo Tokyo Japan; ^3^ Department of Gastroenterological Surgery Tokai University School of Medicine Isehara Japan; ^4^ Division of Gastrointestinal Surgery, Department of Surgery, Graduate School of Medicine Kobe University Kobe Japan; ^5^ Database Committee The Japanese Society of Gastroenterological Surgery Tokyo Japan; ^6^ Department of Frontier Surgery Chiba University Graduate School of Medicine Chiba Japan; ^7^ The Japan Esophageal Society Tokyo Japan; ^8^ Department of Gastroenterological Surgery Osaka University Graduate School of Medicine Osaka Japan; ^9^ Department of Surgery Keio University School of Medicine Tokyo Japan; ^10^ The Japanese Society of Gastroenterological Surgery Tokyo Japan

**Keywords:** anastomotic leak, national clinical database, pneumonia, posterior mediastinal route, retrosternal route, surgical site infection

## Abstract

**Background:**

Esophagectomy followed by gastric conduit reconstruction is a standard surgical procedure for esophageal cancer. However, there is no evidence of the superiority or inferiority of the posterior mediastinal (PM) versus the retrosternal (RS) reconstruction route with regard to short‐term outcomes after esophagectomy. We aimed to elucidate whether the reconstruction route can affect the short‐term outcomes after esophagectomy followed by gastric conduit reconstruction.

**Methods:**

We reviewed the clinical data of patients who underwent esophagectomy between 2016 and 2018 from the Japanese National Clinical Database. This study included 9786 patients who underwent gastric conduit reconstruction through the PM or RS route with cervical anastomosis.

**Results:**

Of the 9786 patients analyzed, 3478 and 6308 underwent gastric conduit reconstruction thorough the PM and RS routes, respectively. The incidence of anastomotic leak and surgical site infection (SSI) was significantly lower in the PM group than in the RS group (11.7% vs 13.8%, *P* = .005 and 8.4% vs 14.9%, *P* < .001, respectively), while the incidence of pneumonia was higher in the PM group (13.7% vs 12.2%, *P* = .040). Generalized estimating equation logistic regression analysis revealed a higher risk of anastomotic leak and SSI (odds ratio [OR], 1.32; 95% confidence interval [CI], 1.15–1.51; *P* < .001 and OR, 2.06; 95% CI, 1.78–2.38; *P* < .001, respectively) and a lower risk of pneumonia (OR, 0.86; 95% CI, 0.75–0.98; *P* = .028) in the RS group than in the PM group.

**Conclusion:**

The findings of this study will help surgeons to design the reconstruction route following esophagectomy.

## Graphical abstract

We reviewed the clinical data of patients who underwent esophagectomy from the Japanese National Clinical Database to elucidate whether the reconstruction route can affect the short‐term outcomes. The retrosternal route was identified as a risk factor for anastomotic leak and surgical site infection, and the posterior mediastinal (PM) route for pneumonia.

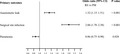

## 1 INTRODUCTION

Esophageal cancer is one of the leading causes of cancer‐related deaths worldwide.^1^ Esophagectomy plays an important role in the multidisciplinary treatment strategies for esophageal cancer.^2^, ^3^, ^4^ In the Asia–Pacific region, including Japan, the vast majority of esophageal cancers are squamous cell carcinomas (SCC) located at the upper to lower thoracic esophagus.^4^, ^5^, ^6^ Surgical treatment of thoracic esophageal SCC generally consists of subtotal esophagectomy, two‐ or three‐field lymphadenectomy, and reconstruction using organs such as the stomach.^6^, ^7^, ^8^ Recent advances in surgical techniques and perioperative management have enabled us to safely perform one‐stage esophagectomy with radical lymphadenectomy followed by reconstruction; however, esophagectomy remains a highly invasive procedure that can lead to severe morbidities such as anastomotic leak, respiratory complications, and cardiac events.^9^, ^10^, ^11^

The stomach is the most commonly used organ for reconstruction following esophagectomy.^3^, ^6^, ^8^ Reconstruction using a gastric conduit can be performed via the posterior mediastinal (PM), retrosternal (RS), or subcutaneous (SC) route, among which PM and RS comprise the vast majority of reconstruction routes due to advantages over the SC route, such as shorter reconstruction route and fewer cosmetic changes after esophagectomy.^6^, ^8^ Esophagogastric anastomosis is generally made at the cervical incision in cases of RS reconstruction. Cervical or intrathoracic anastomosis can be used in cases of PM reconstruction, and cervical anastomosis is commonly performed in several Japanese institutes.^6^ There are some controversies regarding the superiority or inferiority of PM reconstruction to RS reconstruction; however, there is no evidence on the impact of the reconstruction route on short‐term outcomes after esophagectomy based on large‐scale clinical data.

In this study we reviewed the clinical data of 17,478 patients who underwent esophagectomy followed by one‐stage reconstruction using the National Clinical Database (NCD), a nationwide, web‐based, data entry system in Japan.^10^, ^11^, ^12^, ^13^, ^14^ We sought to elucidate whether the reconstruction route can affect the short‐term outcomes after esophagectomy followed by gastric conduit reconstruction.

## 2 MATERIALS AND METHODS

### 2.1 NCD data registration

Details of the data registration system of the Japanese NCD are available elsewhere.^10^, ^11^, ^12^, ^13^, ^14^ The NCD started data registration in January 2011, and ~1,500,000 cases are registered annually from over 5000 institutions, which corresponds to >95% of surgeries in Japan.^14^ For the eight major gastroenterological surgery procedures, including esophagectomy, that were determined to represent the performance of surgery in each specialty, the input of detailed items, such as lab data and operative morbidities, are requested. Since January 2016, more detailed information, including the reconstruction organ, reconstruction route, and the location of anastomosis have been included in the requested items for esophagectomy cases. The Union for International Cancer Control TNM staging version 7 was adopted to classify the pretreatment tumor stages. All variables, definitions, and inclusion criteria for the NCD were accessible online by the participating institutions (http://www.ncd.or.jp/). The NCD supports an E‐learning system that can be used by participants to enter consistent data. The patient variables in the NCD were almost identical to those used by the American College of Surgeons National Surgical Quality Improvement Program (ACS NSQIP).^15^, ^16^

### 2.2 Study design

This was a retrospective cohort study. We reviewed the clinical data of patients who underwent esophagectomy between 2016 and 2018 from the NCD Japan. According to the inclusion criteria, only thoracic esophageal cancer patients who underwent esophagectomy followed by gastric conduit reconstruction through the RS or PM route with cervical anastomosis were included in this study. Patients with metastatic or recurrent diseases, those with clinical T4, TX, NX, or M1 tumors, those who underwent emergency operation, those <18 y, and those with missing data were excluded from this study.

### 2.3 Endpoints

The primary outcome measure was the incidence of major postoperative morbidities, including anastomotic leak; surgical site infection (SSI), including superficial incisional SSI, deep incisional SSI, and organ space SSI with or without leak; and pneumonia. The secondary endpoints included the 30‐d and operative mortality rates, operation time, and bleeding. Operative mortality included all patients who died within the index hospitalization period, regardless of the length of hospital stay (up to 90 d), and any patient who died after hospital discharge (up to 30 d after surgery).

### 2.4 Statistical analysis

For categorical variables, the proportion of patients experiencing the abovementioned outcomes was compared between the PM and RS groups using Pearson's chi‐squared test, and continuous variables were compared using the Mann–Whitney *U*‐test. Considering clustering of the patients at the hospital level, generalized estimating equation (GEE) logistic regression analysis was used for multivariable analysis of the impact of each clinical factor on short‐term outcomes, including age at surgery (<59, 60–64, 65–69, 70–74, 75–79, and ≥80 y), sex (male vs female), body mass index (BMI; <25 vs ≥25 kg/m^2^), weight loss (<10% vs ≥10%), smoking (yes vs no), habitual alcohol use (yes vs no), any respiratory distress (yes vs no), preoperative activities of daily living (ADL) with any assistance (yes vs no), American Society of Anesthesiologists physical status (ASA‐PS; 1–2 vs ≥3), diabetes mellitus (DM) with insulin use (yes vs no), chronic obstructive pulmonary disease (COPD; yes vs no), hypertension (yes vs no), congestive heart failure (yes vs no), previous cardiovascular surgery (yes vs no), previous cerebrovascular disease (yes vs no), need for preoperative dialysis (yes vs no), chronic steroid use (yes vs no), serum albumin level (<2.5 vs ≥2.5 g/dL), creatinine level (<1.2 vs ≥1.2 mg/dL), clinical T stage (T0/Tis/T1a, T1b, T2, T3), clinical N stage (N0, N1, N2, N3), and histological type of cancer (SCC, adenocarcinoma, others). In addition, the use of thoracoscopy or mediastinoscopy for esophagectomy (minimally invasive esophagectomy: MIE), and the hospital esophagectomy case volume according to interquartile ranges (1–8, 9–18, 19–41, ≥42 cases/year) were included in the multivariable analysis based on our previous study.^17^ All statistical tests were two‐sided, and *P* < .05 was considered significant. All statistical analyses were performed using R v. 3.6.3 (2020; R Foundation for Statistical Computing, Vienna, Austria).

## 3 RESULTS

### 3.1 Study population

**FIGURE 1:**
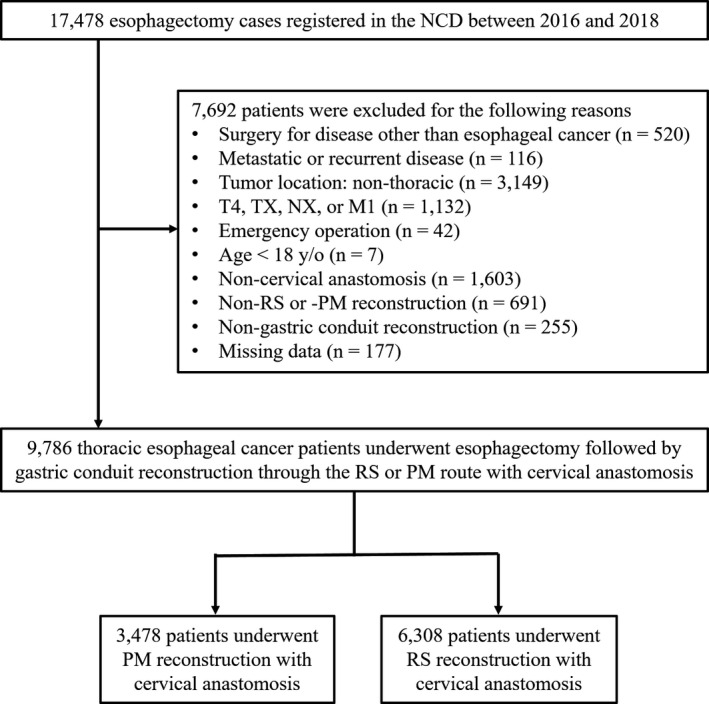
Selection process for the study population

Between January 2016 and December 2018, the NCD registered 17,478 patients who underwent esophagectomy followed by one‐stage reconstruction at Japanese institutes. According to the inclusion and exclusion criteria, 9786 thoracic esophageal cancer patients who underwent esophagectomy followed by gastric conduit reconstruction through the RS or PM route with cervical anastomosis were included for analysis. Of the total analysis population, 3478 (35.5%) and 6308 (64.5%) patients underwent gastric conduit reconstruction thorough the PM route and RS route with cervical anastomosis, respectively (Figure 1).

### 3.2 Risk profile

Of the 9786 thoracic esophageal cancer patients who underwent esophagectomy followed by gastric conduit reconstruction thorough either the PM or RS route with cervical anastomosis, 80.2% were male and 41.8% were elderly patients ≥70 y old. Among these patients, 36.6% had a smoking habit within 1 y, 0.9% needed assistance with ADL, 5.1% demonstrated weight loss of more than 10%, and 7.7% had an ASA‐PS of grade ≥3. Preoperative comorbidities included DM with insulin use in 2.8% of patients, preoperative respiratory distress within 30 d in 0.8%, COPD in 7.6%, and history of cardiac surgery and cerebrovascular disease in 0.6% and 3.0%, respectively. Tumor histology was SCC in 95.1% and adenocarcinoma in 2.3%. Regarding the surgical procedure, MIE was performed in 78.4% of patients. A full demographic and risk profile of the study population is shown in Table 1.

**TABLE 1:** Comparison of preoperative variables between the PM and RS groups

Variables	Total (n = 9786)		PM (n = 3478)		RS (n = 6308)		*P*
	n	%	n	%	n	%	
Age							
<59 y	1675	17.1	546	15.7	1129	17.9	.053
60–64 y	1525	15.6	542	15.6	983	15.6	
65–69 y	2498	25.5	891	25.6	1607	25.5	
70–74 y	2175	22.2	783	22.5	1392	22.1	
75–79 y	1476	15.1	542	15.6	934	14.8	
≥80 y	437	4.5	174	5.0	263	4.2	
Male sex	7850	80.2	2791	80.2	5059	80.2	.976
BMI ≥25	8627	88.2	412	11.8	747	11.8	1
Weight loss, ≥10%	497	5.1	182	5.2	315	5.0	.64
Smoking	3586	36.6	1294	37.2	2292	36.3	.405
Habitual alcohol use	6759	69.1	2361	67.9	4398	69.7	.063
Respiratory distress	74	0.8	32	0.9	42	0.7	.205
ADL, any assistance	92	0.9	41	1.2	51	0.8	.088
ASA‐PS grade ≥3	750	7.7	286	8.2	464	7.4	.133
DM with insulin use	275	2.8	122	3.5	153	2.4	.002
COPD	744	7.6	251	7.2	493	7.8	.303
Hypertension	3824	39.1	1386	39.9	2438	38.6	.253
Congestive heart failure	15	0.2	10	0.3	5	0.1	.024
Past cardiac surgery	55	0.6	42	1.2	13	0.2	<.001
Cerebrovascular disease	296	3.0	113	3.2	183	2.9	.368
Preoperative dialysis	14	0.1	6	0.2	8	0.1	.77
Chronic steroid use	87	0.9	30	0.9	57	0.9	.925
Serum albumin <2.5 g/dL	75	0.8	28	0.8	47	0.7	.838
Serum creatinine ≥1.2 mg/dL	622	6.4	259	7.4	363	5.8	.001
Clinical T stage							
T0, Tis, T1a	1218	12.4	468	13.5	750	11.9	.002
T1b	3242	33.1	1194	34.3	2048	32.5	
T2	1555	15.9	561	16.1	994	15.8	
T3	3771	38.5	1255	36.1	2516	39.9	
Clinical N stage							
N0	4840	49.5	1773	51.0	3067	48.6	.019
N1	2766	28.3	978	28.1	1788	8.3	
N2	1664	17.0	539	15.5	1125	17.8	
N3	516	5.3	188	5.4	328	5.2	
Histology type							
Squamous cell carcinoma	9306	95.1	3311	95.2	5995	95.0	.695
Adenocarcinoma	224	2.3	82	2.4	142	2.3	
Others	256	2.6	85	2.4	171	2.7	
Minimally invasive esophagectomy	7676	78.4	2986	85.9	4690	74.4	<.001
Hospital esophagectomy volume (per year)							
1–8 cases	2168	22.2	873	25.1	1295	20.5	<.001
9–18 cases	2489	25.4	1181	34.0	1308	20.7	
19–41 cases	2637	26.9	1067	30.7	1570	24.9	
≥42 cases	2492	25.5	357	10.3	2135	33.8	

Abbreviations: ADL, activities of daily living; ASA‐PS, American Society of Anesthesiologists physical status; BMI, body mass index; COPD, chronic obstructive pulmonary disease; DM, diabetes mellitus; PM, posterior mediastinal route; RS, retrosternal route.

### 3.3 Background characteristics of the PM and RS groups

Most of the preoperative variables, such as age, BMI, ASA‐PS, and previous cerebrovascular disease were equivalent between the PM and RS groups; however, the rates of DM with insulin use, congestive heart failure, and past cardiac surgery were significantly higher in the PM group than the RS group (3.5% vs 2.4%, *P* = .002; 0.3% vs 0.1%, *P* = .024; and 1.2% vs 0.2%, *P* < .001, respectively) (Table 1). Regarding preoperative lab data, the number of patients with serum creatinine ≥1.2 mg/dl was higher in the PM group than the RS group (7.4% vs 5.8%, *P* = .001) (Table 1). In contrast, clinical T stage and N stage were higher in the RS group than the PM group (*P* = .002 and .019, respectively) (Table 1). There was no significant difference in the histological type of esophageal cancer between the two groups (*P* = .695) (Table 1). MIE was performed more frequently in the PM group than the RS group (*P* < .001) (Table 1). The RS route was more commonly selected for reconstruction after esophagectomy in hospitals with a higher esophagectomy case volume per year (*P* < .001) (Table 1).

### 3.4 Effect of reconstruction route on operation time and bleeding

The median operation time and bleeding in the 9786 patients who underwent esophagectomy followed by gastric conduit reconstruction with cervical anastomosis was 488 (427–549) min and 200 min (100–370) mL, respectively. The operation time was significantly longer in the PM group than in the RS group, although the median value was the same between the two groups (488 [427–610] min vs 488 [366–549] min, *P* < .001). The bleeding volume was lower in the PM group than in the RS group (166 [72–334] ml vs 220 [110–389] ml, *P* < .001) (Table 2).

**TABLE 2:** Comparison of operative outcomes between the PM and RS groups

Variables	Total (n = 9786)	PM (n = 3478)	RS (n = 6308)	*P*
Operation time (median [IQR], min)	488 [427–549]	488 [427–610]	488 [366–549]	<.001
Bleeding (median [IQR], mL)	200 [100–370]	166 [72–334]	220 [110–389]	<.001
Anastomotic leak (%)	1276 (13.0)	408 (11.7)	868 (13.8)	.005
Surgical site infection (%)	1232 (12.6)	293 (8.4)	939 (14.9)	<.001
Pneumonia (%)	1244 (12.7)	475 (13.7)	769 (12.2)	.040
30‐d mortality (%)	60 (0.6)	18 (0.5)	42 (0.7)	.445
Operative mortality (%)	97 (1.0)	33 (0.9)	64 (1.0)	.835

Abbreviations: IQR, interquartile range; PM, posterior mediastinal route; RS, retrosternal route.

### 3.5 Effect of reconstruction route on postoperative morbidities

Major postoperative morbidities, including anastomotic leak, SSI, and pneumonia were observed in 13.0%, 12.6%, and 12.7%, respectively, of the 9786 patients who underwent esophagectomy followed by gastric conduit reconstruction with cervical anastomosis (Table 2). The rates of anastomotic leak and SSI were significantly lower in the PM group than the RS group (11.7% vs 13.8%, *P* = .005 and 8.4% vs 14.9%, *P* < .001, respectively). In contrast, the rate of pneumonia was higher in the PM group than the RS group (13.7% vs 12.2%, *P* = .040) (Table 2).

### 3.6 Mortality

The mortality rates of esophagectomy followed by gastric conduit reconstruction are presented in Table 2. The 30‐d mortality was 0.6%, and the overall operative mortality was 1.0%. There was no significant difference in either 30‐d or operative mortality rates between the PM and RS groups (0.5% vs 0.7%, *P* = .445 and 0.9% vs 1.0%, *P* = .835, respectively).

### 3.7 Risk comparison of postoperative morbidities between the PM and RS groups

**FIGURE 2:**
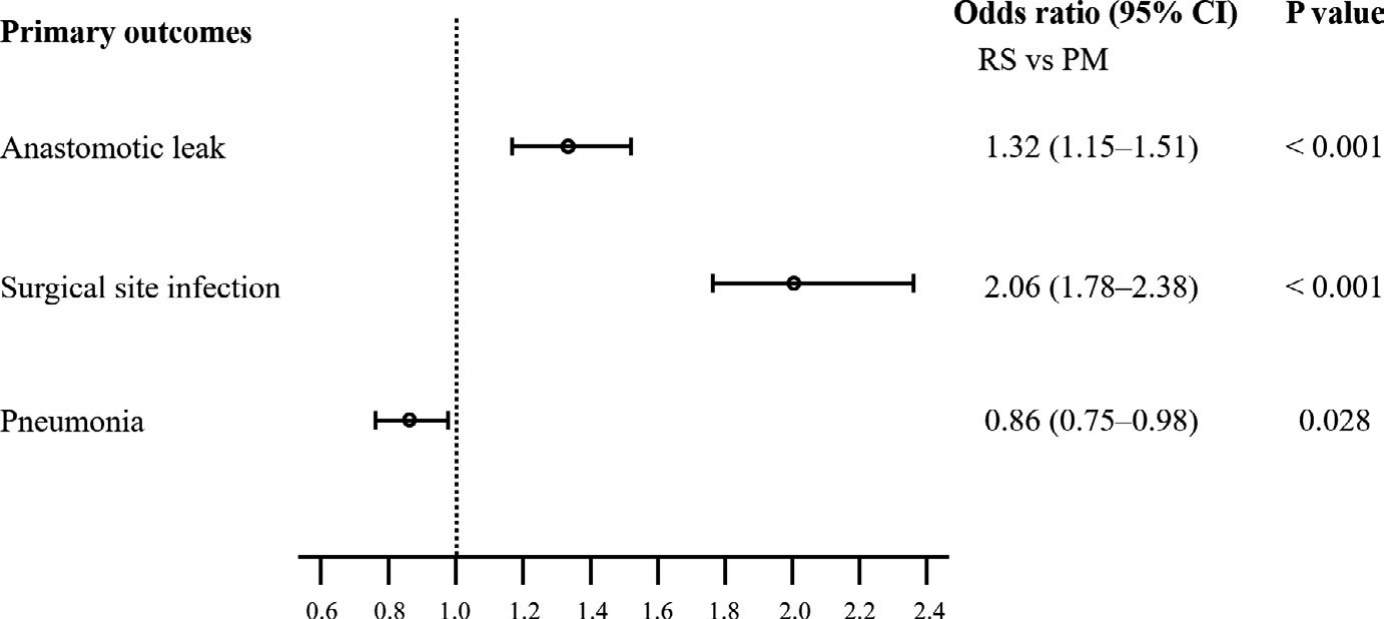
Risk comparison of postoperative morbidities between the posterior mediastinal (PM) and retrosternal (RS) groups. Circles represent the estimate of odds ratio and bars represent their 95% confidence interval (CI)

As shown in Figure 2, GEE logistic regression analysis revealed that the risks of anastomotic leak and SSI were significantly greater in the RS group compared to the PM group (odds ratio [OR], 1.32; 95% confidence interval [CI], 1.15–1.51; *P* < .001, and OR, 2.06; 95% CI, 1.78–2.38; *P* < .001, respectively), whereas the risk of pneumonia was significantly lower in the RS group compared to the PM group (OR, 0.86; 95% CI, 0.75–0.98; *P* = .028).

## 4 DISCUSSION

In this study we analyzed 9786 thoracic esophageal cancer patients in the NCD who underwent esophagectomy followed by gastric conduit reconstruction with cervical anastomosis. Our results demonstrated the RS route as a risk factor for anastomotic leak and SSI and the PM route as a risk factor for pneumonia. To the best of our knowledge, this is the first and largest comparison report based on a nationwide database, to elucidate the impact of reconstruction route on the short‐term outcomes after esophagectomy.

In daily medical practice, the route used as the first choice for reconstruction after esophagectomy is dependent on the institutional policy or surgeons’ preference. However, the final reconstruction route is generally decided based on the tumor stage and the patient's comorbidities, such as DM, which is a known risk factor for anastomotic leak and SSI,^11^, ^18^ and cardiac dysfunction, which may be affected by the stomach in the RS route.^19^, ^20^ The history of cardiac surgery can make it difficult to construct the RS route through the front of the heart. In cases of local recurrence following PM reconstruction, tumors may invade the gastric conduit, leading to secondary complications, such as bleeding and stenosis, and difficulty in radiation therapy.^21^ These general backgrounds in deciding the reconstruction route were reflected in the present study as the difference between the PM and RS groups is shown in Table 1.

In previous reports using NCD data, anastomotic leak and SSI were the most frequent surgical complications (approximately 13% and 14%, respectively), and pneumonia was the most frequent nonsurgical complication (approximately 15%) among major morbidities after esophagectomy.^10^, ^11^, ^22^ In the present study, the rates and risks of anastomotic leak, SSI, and pneumonia were investigated as major morbidities after esophagectomy, as well as candidates of reconstruction route‐related morbidities clinically.

Previous studies, including one randomized control trial (RCT) and five retrospective studies that enrolled a relatively small number of patients, have compared postoperative morbidities between the PM and RS routes in patients undergoing esophagectomy followed by gastric conduit reconstruction with cervical anastomosis.^21^, ^23^, ^24^, ^25^, ^26^, ^27^ Whereas two retrospective studies showed a higher incidence of anastomotic leak in the RS group,^24^, ^25^ the others showed no significant differences between the PM and RS routes,^21^, ^23^, ^26^, ^27^ and the impact of reconstruction route on anastomotic leak remained controversial. In the present study, anastomotic leak and SSI were more frequently observed in the RS group than the PM group, and multivariable analyses identified the RS route as an independent risk factor for anastomotic leak and SSI. The higher rate and risks of SSI appear to be affected by the higher incidence of anastomotic leak in the RS group. However, the mechanisms by which the RS route increased the risk of anastomotic leak are currently unclear. Although the length of reconstruction route may explain the risk of anastomotic leak, there are controversies surrounding the required conduit length between the PM and RS routes.^28^, ^29^, ^30^, ^31^ Furthermore, the size of the thoracic inlet (superior thoracic aperture) is reportedly associated with cervical anastomotic leak, both in the PM and RS reconstructions, suggesting that compression of the clavicular head and the sternoclavicular joint may impair blood flow of the gastric conduit.^32^, ^33^, ^34^ However, it is unclear which route is more significantly affected by a narrow thoracic inlet, and whether this may lead to different risks of anastomotic leak between the two groups. The effect of the gastric conduit width (wide vs narrow) on anastomotic leak is another topic of clinical interest; however, there is currently no consensus on the superiority of either method.^35^, ^36^, ^37^, ^38^, ^39^ Although the NCD does not request information on the width of gastric conduit, the rate of reconstruction using a whole stomach should be very low (about 1%) according to the comprehensive registry of esophageal cancer in Japan.^6^

Regarding pneumonia, there was no significant difference between the PM and RS routes in previous studies.^21^, ^23^, ^24^, ^25^, ^26^, ^27^ Therefore, for the first time, the present study showed the lower risks of pneumonia in the RS route over the PM route after esophagectomy followed by gastric conduit reconstruction using a Japanese nationwide database. In this study, the slightly higher rate of elderly patients ≥70 y in the PM group than the RS group (43.1% vs 41.1%) may explain the cause of higher incidence of pneumonia; however, multivariable analyses identified the PM route as an independent risk factor for pneumonia. In the PM group, the posterior mediastinum could be massively occupied by the pulled‐up stomach and omentum, depending on the size of the gastric conduit and the abdominal visceral fat volume; this may compress the trachea and bronchus, and cause pulmonary atelectasis and pneumonia. In addition, there may be a difference between the PM and RS groups with regard to the incidence of gastroesophageal reflux, which may also cause pneumonia.^40^ Because anastomotic leak may be one of the multiple causes of postoperative pneumonia, a possible higher impact of anastomotic leak on the development of pneumonia in the PM group may explain the higher incidence of pneumonia in the PM group than in the RS group. However, it is difficult to differentiate which complication occurred first in patients with a coincidence of anastomotic leak and pneumonia, and the causal relationship between these two complications remain unclear in this study using the NCD. These hypothetical mechanisms need to be verified in future studies, including more detailed data, such as body fat percentage, acid reflux, and the onset time of complications.

Regarding mortality after esophagectomy, there was no significant difference in either 30‐d or operative mortality rates between the PM and RS groups. Although the effects of the reconstruction route on the long‐term outcomes were not evaluated, the difference in the major morbidity rates between the PM and RS routes in the present study suggest an impact of the reconstruction route on the oncological outcomes after esophagectomy. Indeed, previous studies on esophageal SCC and adenocarcinoma showed an association of postoperative complications such as pneumonia with poor oncological outcomes.^41^, ^42^, ^43^ Therefore, the reconstruction route following esophagectomy may impact survival by affecting the risks of postoperative morbidities.

The limitations of the current study include a lack of anastomotic procedures, such as hand‐suture and stapling anastomosis, and a lack of long‐term outcomes over 3 mo and quality of life after surgery. In addition, our study did not capture known short‐ and long‐term complications, such as anastomotic stenosis, gastroesophageal reflux, and malnutrition because the NCD did not collect these data. The raw data outcomes of anastomotic leak and SSI could be underestimated, and those of pneumonia could be overestimated by hospital volume, which may be associated with postoperative complications, as speculated by its impact on risk‐adjusted mortality following esophagectomy^44^ and the preference of the RS route for reconstruction in hospitals with a higher esophagectomy case volume per year (Table 1). Training status and compliance, and the certification status of the institute and surgeon could also influence the outcomes.^45^ However, these factors were included in the multivariable analysis as a hospital esophagectomy case volume in this study, and the outcomes shown in Table 2 were further supported by the multivariable analysis.

A major strength of this study is that this is a large‐scale comparison report based on a nationwide database. In contrast to RCTs, which generally enroll patients below a certain age limit, without serious comorbidities, in limited institutions the NCD registered almost all surgical cases in Japan. The present study analyzed a large number of patients who underwent esophagectomy in the whole of Japan using the NCD, and unveiled the current status of reconstruction route choice after esophagectomy. Moreover, we examined the impact of the reconstruction route on postoperative morbidities after esophagectomy, without excluding elderly patients or those with serious comorbidities.

In conclusion, the present study first analyzed the impact of the reconstruction route on postoperative morbidities using the nationwide clinical database of patients undergoing esophagectomy for esophageal cancer. The RS route was identified as a risk factor for anastomotic leak and SSI, and the PM route was identified as a risk factor for pneumonia after esophagectomy. Although there may be some bias in the indication of reconstruction route based on the patients’ condition and tumor stage, risk‐adjusted models in the present study provided important information about the risks of major morbidities, which should be considered for the indication of reconstruction route preoperatively.

## DISCLOSURE

Conflict of interest: Hideki Endo, Hiroyuki Yamamoto, and Hiroaki Miyata are affiliated with the Department of Healthcare Quality Assessment at the University of Tokyo. The other authors have no conflicts of interest.

Ethical approval: The protocol for this research project was approved by the Ethics Committee of Hamamatsu University School of Medicine (Approval number: 19‐102), and it conforms to the provisions of the Declaration of Helsinki. The opt‐out method to obtain patient consent was utilized at each institution.
